# Hyperspectral Imaging as an Early Biomarker for Radiation Exposure and Microcirculatory Damage

**DOI:** 10.3389/fonc.2015.00232

**Published:** 2015-10-26

**Authors:** Michael S. Chin, Brian B. Freniere, Luca Lancerotto, Jorge Lujan-Hernandez, Jonathan H. Saleeby, Yuan-Chyuan Lo, Dennis P. Orgill, Janice F. Lalikos, Thomas J. Fitzgerald

**Affiliations:** ^1^Department of Radiation Oncology, University of Massachusetts Medical School, Worcester, MA, USA; ^2^Division of Plastic Surgery, Brigham and Women’s Hospital, Boston, MA, USA; ^3^Division of Plastic Surgery, University of Massachusetts Medical School, Worcester, MA, USA

**Keywords:** hyperspectral imaging, diffuse reflectance imaging, microcirculatory damage, radiation exposure, radiation dosage, radiation effects, perfusion imaging

## Abstract

**Background:**

Radiation exposure can lead to detrimental effects in skin microcirculation. The precise relationship between radiation dose received and its effect on cutaneous perfusion still remains controversial. Previously, we have shown that hyperspectral imaging (HSI) is able to demonstrate long-term reductions in cutaneous perfusion secondary to chronic microvascular injury. This study characterizes the changes in skin microcirculation in response to varying doses of ionizing radiation and investigates these microcirculatory changes as a possible early non-invasive biomarker that may correlate with the extent of long-term microvascular damage.

**Methods:**

Immunocompetent hairless mice (*n* = 66) were exposed to single fractions of superficial beta-irradiation in doses of 0, 5, 10, 20, 35, or 50 Gy. A HSI device was utilized to measure deoxygenated hemoglobin levels in irradiated and control areas. HSI measurements were performed at baseline before radiation exposure and for the first 3 days post-irradiation. Maximum macroscopic skin reactions were graded, and histological assessment of cutaneous microvascular densities at 4 weeks post-irradiation was performed in harvested tissue by CD31 immunohistochemistry.

**Results:**

CD31 immunohistochemistry demonstrated a significant correlation (*r* = 0.90, *p* < 0.0001) between dose and vessel density reduction at 4 weeks. Using HSI analysis, early changes in deoxygenated hemoglobin levels were observed during the first 3 days post-irradiation in all groups. These deoxygenated hemoglobin changes varied proportionally with dose (*r* = 0.98, *p* < 0.0001) and skin reactions (*r* = 0.98, *p* < 0.0001). There was a highly significant correlation (*r* = 0.91, *p* < 0.0001) between these early changes in deoxygenated hemoglobin and late vascular injury severity assessed at the end of 4 weeks.

**Conclusion:**

Radiation dose is directly correlated with cutaneous microvascular injury severity at 4 weeks in our model. Early post-exposure measurement of cutaneous deoxygenated hemoglobin levels may be a useful biomarker for radiation dose reconstruction and predictor for chronic microvascular injury.

## Background

Exposure to ionizing radiation can have profound biological consequences. Scenarios for radiation exposure range from external beam radiotherapy for oncologic treatment to uncontrolled release events such as a nuclear attack or accident. The disasters at Fukushima and Chernobyl emphasize the real possibility of the latter scenario. Cutaneous skin reactions in response to ionizing radiation exposure are major consequences in both cases. These skin reactions in the acute phase can range from mild skin erythema to ulceration. For radiotherapy alone, it has been reported that a majority of patients receiving radiotherapy experience some degree of acute skin reaction (1).

The precise etiology of ionizing radiation-induced skin injury remains unclear. Multiple theories proposed include direct cellular injury, cell signaling dysregulation, and ischemia (2–4). Recently, the literature has suggested that cutaneous ischemia may be the predominant characteristic that links both acute and chronic-phase injuries (5, 6). However, these studies have all been limited as they use only a single large dose of ionizing radiation.

In our laboratory, we have established a reliable model of radiation-induced skin injury using a hairless mouse and a single fixed-dose of surface beta-irradiation (7, 8). Using hyperspectral imaging (HSI) technology, we have identified characteristic changes in cutaneous perfusion that are reproducible in magnitude and in timing, preceding the visible appearance of skin injury, and corresponding to the degradation of the dermal vascular density.

Two significant issues remain unaddressed: (1) the relationship between the dose of cutaneous irradiation and the magnitude of cutaneous perfusion changes observed over time and (2) the relationship between early cutaneous perfusion changes and the development of acute or chronic skin injury post-irradiation. A better understanding of the latter may have significant clinical application since the development of acute skin reactions during radiotherapy may necessitate a treatment break. It has been estimated that a treatment break of more than a week during breast cancer radiotherapy can negatively impact recurrence rate and overall survival (9, 10). Therefore, the ability to detect and predict high-risk areas for developing skin damage and individualize the treatment plan accordingly may prevent the need for these detrimental treatment breaks.

## Materials and Methods

### Animals and Irradiation Procedure

All handling of and procedures performed with animals were done in accordance with a protocol (UMass IACUC Protocol #A2354) approved by our Institutional Animal Care and Use Committee. Prior to this experiment, we established a model of acute radiation-induced skin injury (7). The radiation source utilized was a Strontium-90 beta-emitter with an active diameter of 9 mm, which created an 8 mm diameter skin injury. This beta-emitter source delivered less than 10% of the total dose deeper than 3.6 mm into the skin, avoiding injury to internal organs.

Hairless, immunocompetent adult mice (SKH-1 Elite, Charles River Laboratories, Wilmington, MA, USA) were used. Mice (*n* = 66) were equally divided into six groups of eleven mice each. Each group received one of six pre-specified doses of ionizing radiation respectively: 0, 5, 10, 20, 35, and 50 Gy.

On day 0, mice were anesthetized. Tattooing was performed bilaterally on the dorsal flank skin of the mice to act as fiducial marks. Cutaneous perfusion was assessed using HSI prior to irradiation as described below. This pre-irradiation assessment served as a baseline level for subsequent comparison. Mice then received a single pre-specified dose of irradiation as described above. Accurate dose-delivery was ensured prior to animal irradiation using radiochromic film dosimetry. Following irradiation, mice were recovered and housed individually.

### Hyperspectral Imaging for Evaluation of Cutaneous Perfusion

Evaluation of cutaneous perfusion was accomplished using a novel application of an existing technology, HSI. HSI is a method of wide-field diffuse reflectance spectroscopy that utilizes a spectral separator to vary the wavelength of light entering a digital camera and provides a diffuse reflectance spectrum for every pixel. These spectra are then compared to standard transmission solutions to calculate the concentration of deoxy-hemoglobin (DeoxyHb) in each pixel, from which spatial maps of these parameters are constructed.

Using HSI, cutaneous perfusion was analyzed over the first 3 days after radiation exposure. During this time, HSI measurements were performed in all groups, once daily per mouse, for a total of 198 image acquisitions. Skin reactions were then evaluated twice weekly for the first 14 days and then weekly through 28 days post-irradiation. At the time of each evaluation, mice were anesthetized and maintained at standard body temperature. The OxyVu-2 device (HyperMed, Greenwich, CT, USA) was utilized for HSI acquisitions. The OxyVu-2-generated spatial maps of tissue DeoxyHb were used for quantification of cutaneous perfusion. These maps were analyzed with MATLAB R2010b (Mathworks Inc., Natick, MA, USA). Mean values of DeoxyHb were calculated for a 79-pixel area corresponding to the irradiated area on each flank. This 79-pixel area was determined precisely over time with reference to the fiducial tattoo marks that were placed prior to irradiation.

Similarly, areas of non-irradiated contralateral flank skin were quantified to ensure that any changes in perfusion observed in the irradiated skin were not due to natural variations or systemic phenomena.

Values for DeoxyHb parameters for post-irradiation time points are expressed as relative to pre-irradiation values within the same area of skin. Mean relative values reported hereafter reflect the average of relative levels for all subjects within a dose group at a specified time point.

### Post-Irradiation Tissue Analysis

On day 28 post-irradiation, mice were euthanized following cutaneous perfusion assessment. Immediately post-mortem, irradiated and non-irradiated skin from both flanks was harvested. Tissue was fixed en bloc in 10% neutral-buffered formalin solution and kept at 4°C overnight for paraffin embedment. Paraffin-embedded sections were re-hydrated though a decreasing alcohol series and stained for vasculature as described previously (8). Primary antibody (PECAM-1) for vasculature staining (BD Pharmingen, San Jose, CA, USA) was incubated at 4°C overnight. Signals were intensified by using a tyramide amplification system (PerkinElmer, Boston, MA, USA) and activated with DAB Chromogen (Dako North America Inc., Carpinteria, CA, USA). Slides were counterstained with hematoxylin.

Digital images were obtained from the center of all stained skin sections at 10× magnification with an Olympus BX53 microscope (Olympus America Inc., Center Valley, PA, USA). Two-blinded reviewers quantified vessel density using three random standard-sized fields and results were averaged. Vessel densities for each animal were expressed as a ratio between the irradiated sample and its respective contralateral non-irradiated internal control, yielding normalized mean vessel densities.

### Statistical Analysis

For hyperspectral data, plots of perfusion data are expressed as the relative mean unless otherwise specified. Changes in DeoxyHb and dose were correlated with cutaneous blood vessel densities using linear regression models. Statistical significance was assumed for *p* values <0.05.

## Results

### Macroscopic Skin Reaction

Mice were irradiated as previously described without complication. Mice gained weight appropriately following irradiation and no morbidity or mortality was observed. Skin reactions began forming in all groups by approximately 1 week post-irradiation. Maximal skin reactions were observed by day 14 in all groups and scored using the Radiation Therapy Oncology Group toxicity scoring system, which describes skin reactions from erythema to ulceration over a four-point scale (11). Maximal skin reactions were observed to be characterized by erythema in the 5 and 10 Gy groups. Dry desquamation was observed as the maximal skin reaction of mice receiving 20 and 35 Gy. In the 50 Gy group, moist desquamation was observed in all irradiated areas (Table 1).

**Table 1:** **Maximal skin reaction scores (0–4) at 14 days post-irradiation**.

Dose (Gy)	0	5	10	20	35	50
Skin reaction	0 (11)	1 (11)	1 (11)	2 (11)	2 (11)	3 (11)

### Changes in Microvascular Density

**Figure 1:**
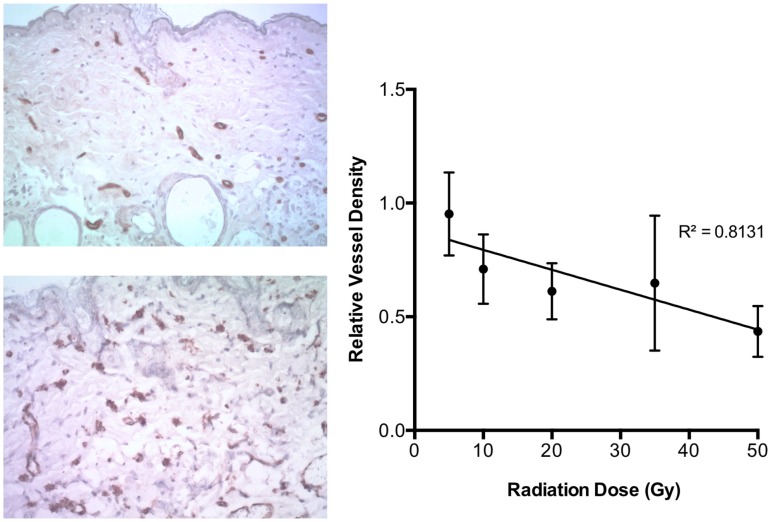
**On left, examples of CD31 staining in skin exposed to 50 Gy (top) and unirradiated (bottom)**. On right, relative vessel density at 4 weeks after radiation dose demonstrated an inverse linear relationship with initial radiation dose exposure (*r* = 0.90, *p* < 0.0001).

A pronounced reduction in cutaneous vascular densities at 4 weeks following irradiation was observed. CD31 immunohistochemistry demonstrated an inverse correlation (*r* = 0.90, *p* < 0.0001) between dose and vessel reduction severity, with vessel counts decreasing as the dose increased (Figure 1).

### Relationship of Early Deoxygenated Hemoglobin Changes and Radiation Dose

**Figure 2:**
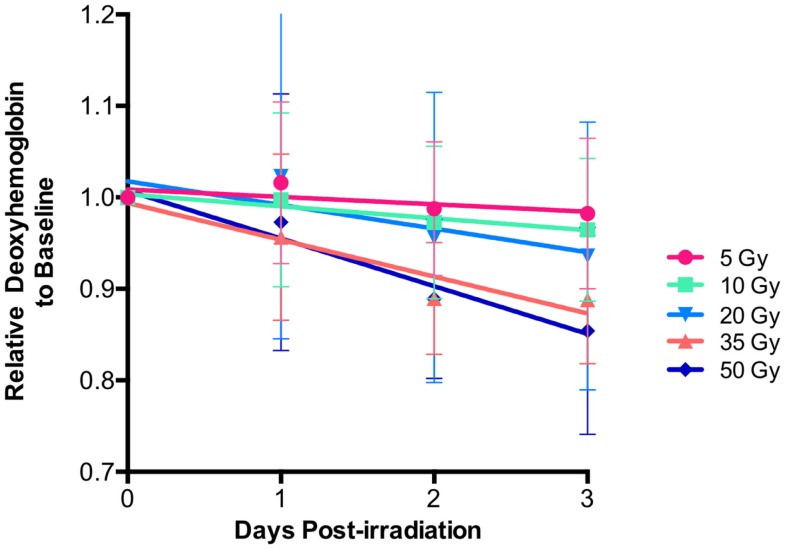
**Relative deoxy-hemoglobin changes decreased over the first 3 days for each level of radiation exposure**. Note the increasing rate of change as the radiation dose becomes larger.

**Figure 3:**
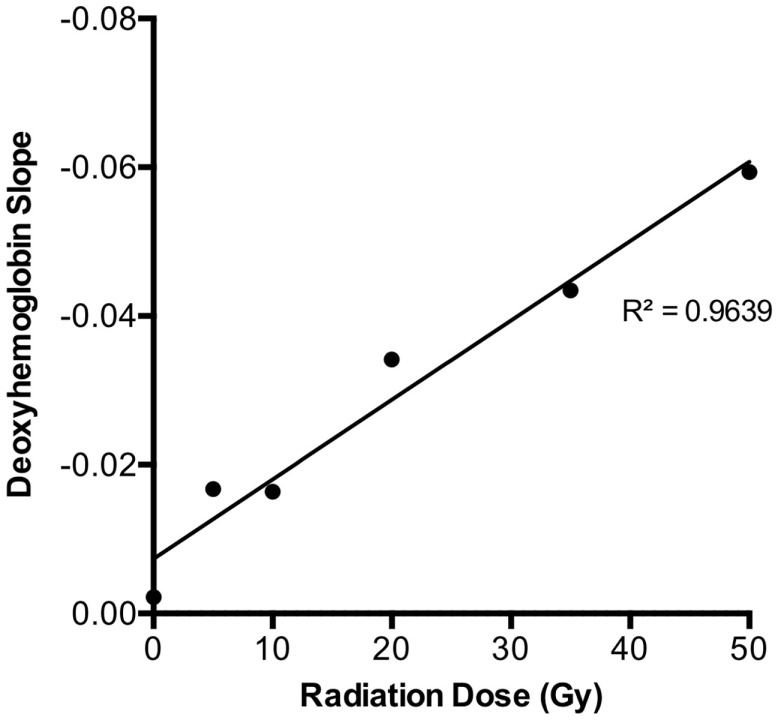
**Deoxy-hemoglobin trend over the first 3 days for each dose level revealed a strong linear relationship (*r* = 0.98, *p* < 0.0001)**. As this plot was derived from the average slope of each dose group depicted in Figure 2 (total *n* = 66), SDs were not plotted.

Using HSI analysis, early changes in deoxygenated hemoglobin levels were observed during the first 3 days post-irradiation in all groups. An acute decrease in deoxygenated hemoglobin was seen for each dose over the first 3 days before any visible skin reactions were observed (Figure 2). When further examining the behavior of deoxygenated hemoglobin over the first 3 days, it was noted that the slope changed linearly with increasing radiation dose exposure (*r* = 0.98, *p* < 0.0001) (Figure 3).

### Correlation between Early Deoxygenated Hemoglobin Changes and Microvascular Density

**Figure 4:**
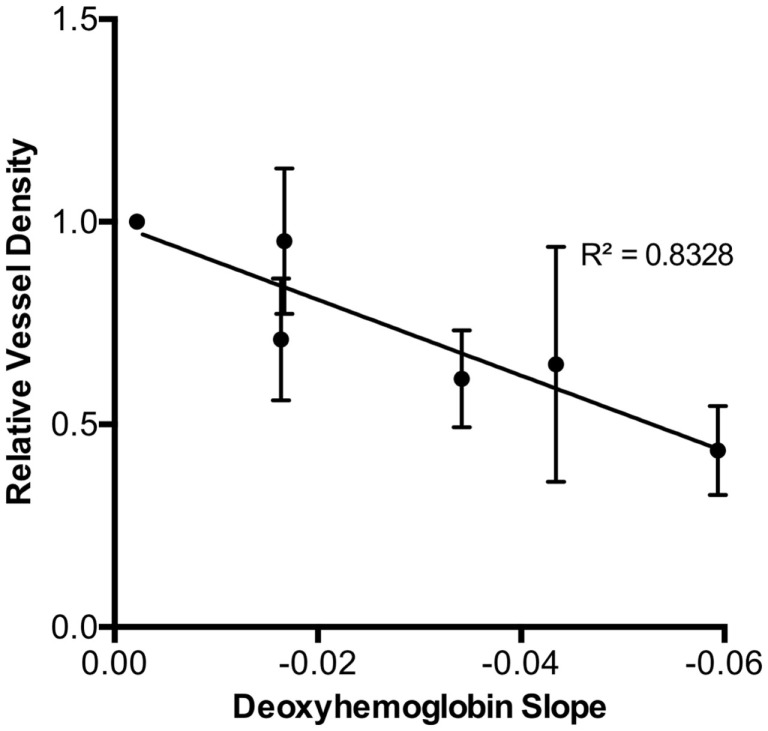
**The final 4-week relative vessel density demonstrated a strong inverse relationship with initial 3-day deoxy-hemoglobin slope for each radiation dose (*r* = 0.91, *p* < 0.0001)**.

Using the relationships between early DeoxyHb changes with dose, we could also observe that deoxygenated hemoglobin decrease at 3 days had a strong linear association with microvascular density by 4 weeks for the given doses, indicating that early deoxygenated hemoglobin response could be predictive of final degree of microvascular damage. There was a highly significant correlation (*r* = 0.91, *p* < 0.0001) between these early changes in deoxygenated hemoglobin and late vascular injury severity assessed (Figure 4).

### Relationship between Early Deoxygenated Hemoglobin Changes and Skin Reaction

**Figure 5:**
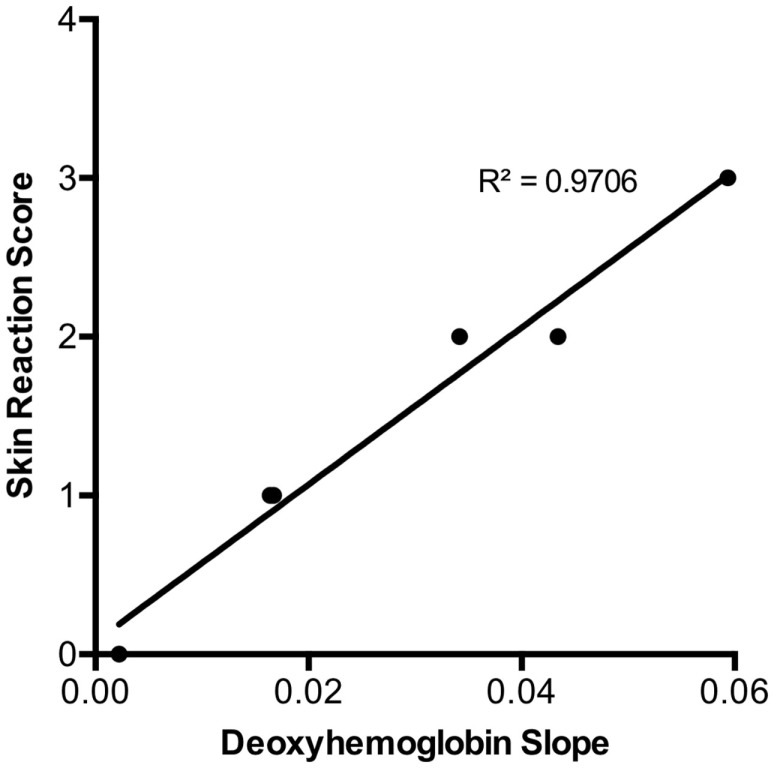
**Maximal skin reaction score by day 14 was strongly correlated to the initial three day deoxy-hemoglobin slope for each radiation dose (*r* = 0.98, *p* < 0.0001)**. There were no SDs of the skin reaction scores plotted as there was no categorical variation within each group (total *n* = 66) as illustrated in Table 1.

In addition, when examining the relationship between deoxygenated slope change in the first 3 days and maximal skin reaction at 14 days, there was a strong linear correlation (*r* = 0.98, *p* < 0.0001) (Figure 5). This suggests that early decreases seen during serial assessment of deoxygenated hemoglobin may be related to the degree of maximal skin reaction after radiation exposure.

## Discussion

Exposure to ionizing radiation is known to induce the expression of numerous vasoactive mediators (12), as well as directly injure the cutaneous microvasculature (2). These effects have the likely consequence of altering cutaneous perfusion. However, little is known about the relationship between the degree of alteration in cutaneous perfusion following exposure and the varying doses of ionizing radiation received. This study was designed to better characterize changes in microcirculation over wide range of superficial radiation exposure.

This project highlights our ability to accurately deliver varying doses of ionizing radiation to the superficial soft tissues using our established model (7, 8). We were able to achieve reproducible results with no off-target morbidity, per previous investigations. The delivery of doses of a much smaller magnitude than our previously described 50 Gy dose allows us to further investigate the effects of fractionated irradiation schemes on cutaneous perfusion. This will be particularly important in translating our work closer toward a model that reflects the low dose fractionation schemes currently employed in therapeutic external beam radiation.

Our previous studies have demonstrated that HSI can be used to analyze acute changes in perfusion and oxygenation in the first week after exposure to radiation. In particular, our initial study suggested a sharp decline in deoxy-hemoglobin within the first several days (7). The current study elaborates on these findings suggesting that increasing radiation doses correlate to deoxygenated hemoglobin response. This phenomenon may be explained by the acute vasoactive effects of radiation, as previous work has shown increased vascular permeability after exposure to increasing amounts of radiation. Krishnan et al. demonstrated using a radiolabeled technique that albumin extravasation into the interstitial space was dose-related (13, 14). In line with these findings, our observed changes in intravascular deoxygenated hemoglobin using HSI may be a result of this dose-dependent phenomenon, making this study the first to demonstrate a non-invasive biomarker of radiation.

Detection of radiation exposure or injury by rapid and non-invasive means is currently unavailable. To date, numerous strategies that minimize time and invasiveness have been proposed to perform biodosimetry. Some of these strategies include utilization of serum biomarkers, and currently, the gold standard for detection of radiation exposure is chromosomal analysis (15). However, since chromosomal analysis requires venipuncture and need for a laboratory setting to process samples, it is unable to provide point-of-care results for triage or treatment guidance. Serum biomarkers present an opportunity to correct some of these shortcomings by allowing for potential point-of-care testing without the need for a laboratory setting (16). However, at present, there are no validated serum biomarkers in clinical use that provide sensitive and specific assessments of radiation exposure and biodosimetry despite numerous potential targets being identified (17–23). In addition, serum biomarkers are still invasive, requiring venipuncture. Non-invasive monitoring of deoxygenated hemoglobin may provide a fast and reliable method for biodosimetry and post-exposure dose reconstruction.

Other studies focused primarily on isotope flow markers to measure the extent microvascular change, demonstrated that exposures varied according to dose. However, no studies histologically examined vessel density correlation with dose exposure (13, 14, 24, 25).

The current study demonstrated that over a large exposure gradient, the initial radiation dose appears to be directly correlated to the degree of microvascular injury. This finding is consistent with results by Haubener et al. who showed that varying doses of single fraction radiation exposure had an increasing effect on diminishing endothelial cell number *in vitro* (26).

Furthermore, we found that a strong relationship exists between deoxygenated hemoglobin response and final vessel density after radiation exposure. This suggests the possibility of using early monitoring of deoxygenated hemoglobin for prediction of chronic vascular damage.

In parallel, our results also suggest that deoxygenated hemoglobin response may be related to formation of acute skin reaction seen weeks after initial radiation exposure. No previous studies have been able to identify a biomarker that predicts the formation of an adverse skin reaction after exposure to radiation. If supported by clinical studies, applications of this finding could result in improved skin monitoring of patients receiving both therapeutic and diagnostic radiologic procedures. Currently, the development of acute skin reactions during radiotherapy usually signals a treatment break. It has been estimated that a treatment break of a more than a week during breast cancer radiotherapy can negatively impact recurrence rate and overall survival (9, 10). Therefore, the ability to detect and predict high-risk areas or high-risk patients for developing skin reactions earlier and change the treatment plan accordingly, may prevent the need for these detrimental treatment breaks.

The current feasibility study suggests that changes in hemoglobin may be assessed in stereotactic body radiosurgery procedures where a single fraction of high dose (30 Gy or higher) may be used in radiation treatment (27, 28). Single high dose radiation exposures may also occur during fluoroscopically guided procedures. Patients undergoing these procedures can receive relatively high radiation doses, which have been documented as resulting in acute and chronic skin injury (29). One major limitation of the current study is the inability to make any clinical inferences regarding deoxygenated hemoglobin response after multiple fractions of radiation that are seen in more traditional oncologic radiation therapy. We are actively pursuing this clinical research in fractionated therapy, and preliminary data has indicated radiation dose can be predicted through a mathematical model using HSI as a metric.

## Conclusion

The current study suggests that early measurement of cutaneous deoxygenated hemoglobin levels after radiation exposure may be a useful biomarker for dose reconstruction and also for chronic microvascular injury. Changes in deoxygenated hemoglobin may also be correlated to acute skin reactions before any are visible. Clinical studies are needed to validate the relationship between early changes in cutaneous circulation following irradiation and subsequent injury development.

## Conflict of Interest Statement

The authors declare that the research was conducted in the absence of any commercial or financial relationships that could be construed as a potential conflict of interest.
